# Prevalence of complaints of arm, neck and shoulder among computer office workers and psychometric evaluation of a risk factor questionnaire

**DOI:** 10.1186/1471-2474-8-68

**Published:** 2007-07-14

**Authors:** Shahla Eltayeb, J Bart Staal, Janneke Kennes, Petra HG Lamberts, Rob A de Bie

**Affiliations:** 1 Maastricht University, Department of Epidemiology and Caphri Research Institute, Maastricht, The Netherlands; 2 Ahfad University for Women, School of Psychology and Preschool Education, Omdurman, Sudan; 3 Lievensberg Hospital, Emergency Department, Bergen op Zoom, The Netherlands; 4 Public Health Service South Limburg, Geleen, The Netherlands

## Abstract

**Background:**

Complaints of Arm Neck and Shoulder (CANS) represent a wide range of complaints, which can differ in severity from mild, periodic symptoms to severe, chronic and debilitating conditions. They are thought to be associated with both physical and psychosocial risk factors. The measurement and identification of the various risk factors for these complaints is an important step towards recognizing (a) high risk subgroups that are relevant in profiling CANS; and (b) also for developing targeted and effective intervention plans for treatment. The purpose of the present study was to investigate the prevalence of CANS in a Dutch population of computer workers and to develop a questionnaire aimed at measuring workplace physical and psychosocial risk factors for the presence of these complaints.

**Methods:**

To examine potential workplace risk factors for the presence of CANS, the Maastricht Upper Extremity Questionnaire (MUEQ), a structured questionnaire, was developed and tested among 264 computer office workers of a branch office of the national social security institution in the Netherlands. The MUEQ holds 95 items covering demographic characteristics, in addition to seven main domains assessing potential risk factors with regard to (1) work station, (2) posture during work, (3) quality of break time, (4) job demands, (5) job control, and (6) social support. The MUEQ further contained some additional questions about the quality of the work environment and the presence of complaints in the neck, shoulder, upper and lower arm, elbow, hand and wrist. The prevalence rates of CANS in the past year were computed. Further, we investigated the psychometric properties of the MUEQ (i.e. factor structure and reliability).

**Results:**

The one-year prevalence rate of CANS indicated that 54% of the respondents reported at least one complaint in the arm, neck and/or shoulder. The highest prevalence rates were found for neck and shoulder symptoms (33% and 31% respectively), followed by hand and upper arm complaints (11% to 12%) and elbow, lower arm and wrist complaints (6% to 7%). The psychometric properties of the MUEQ were assessed using exploratory factor analysis which resulted in the identification of 12 factors. The calculation of internal consistency and cross validation provided evidence of reliability and lack of redundancy of items.

**Conclusion:**

Neck and shoulder complaints are more frequently reported among Dutch computer workers than arm, elbow and hand complaints. The results further indicate that the MUEQ has satisfactory reliability and internal consistency when used to document CANS among computer workers in the Netherlands.

## Background

Complaints of arm, neck and shoulder (CANS) were recognized in the early seventies as an important cause of work disability. They were introduced as "occupational cramps" or "occupational myalgia" and suspected of being associated with numerous occupations and work activities [1]. The rapid development of information technology has entailed changes in working life during the recent decades. It is estimated that more than half of the working population in Western societies currently use personal computers at work [2]. Additionally, the relative time spent in front of the computer and the use of a computer mouse has increased rapidly over the years. It is likely that these developments may have contributed to the increasing burden of CANS [3].

The term CANS is introduced in the Netherlands and indicates "musculoskeletal complaints of arm, neck and/or shoulder not caused by acute trauma or by any systemic disease" [4]. CANS may cause severe and debilitating symptoms such as pain, numbness, and tingling. It may further result in reduced worker productivity, inability to perform job tasks and an increase in workers compensation costs [1]. In the Netherlands alone the incidences of CANS vary between 20 and 40% a year [5]. In the USA, the Bureau of Labour Statistics estimated that in 1996 64% of all new work-related disability cases were related to CANS [6]. Research efforts on the identification of risk factors for the development of work-related CANS [5,7], has shown that these complaints may not be caused solely by high physical job demands (such as repetitive movements, awkward posture etc.) but also by psychosocial demands (such as low social support, high job demands etc.) [8]. At the current stage, the exact relationship between these risk factors and the development CANS is still not clear [9] and more research is needed.

The identification of risk factors for the development of CANS before they develop into a disabling musculoskeletal complaints is an important step in order to recognize relevant subgroups who have a high risk profile for CANS and also, in the longer run, to develop targeted and effective screening and interventions. Hence, targeting computer workers as the selected case population on which to base and to develop measurement tools specifying the risk factors of CANS would seem the appropriate first step.

Although there is an impressive body of literature with regard to measurement tools [10-12], nevertheless there is a need for developing a non complicated yet comprehensive questionnaire for the assessment of CANS [13]. We selected several items related to the presence of CANS from the Karasek Job Content Questionnaire (JCQ) which had been used already in other studies on risk factors for upper extremity complaints [14-16]. Additional, items were selected from the standardised Dutch Musculoskeletal Questionnaire (DMQ) [10,17]. The current study aims to present a questionnaire that would address the occurrence, nature and possible work-related physical and psychological risk factors of CANS among computer users with known psychometric properties. For this purpose a population of Dutch office workers was chosen. This paper presents the one-year prevalence rates of CANS in this working population and the psychometric properties (i.e. factor structure and reliability) of this questionnaire.

## Methods

### Study population and data collection

Study data were derived from a longitudinal cohort study which was conducted in 2002 among 600 office workers at the GAK (National Social Security Institution). Workers who perform computer office tasks (i.e. administrative, graphical and data entry tasks) were invited to participate in the study. The GAK Netherlands is a public benefits agency for statutory regulations in the field of sickness, incapacity for work and unemployment. The GAK carries out a number of national social insurances. It has several branches in the country of which two were selected in Maastricht and Heerlen, both towns in the south-east region of the Netherlands. Data were collected with self-administered postal questionnaires. In mid December 2001 the questionnaires were distributed by internal mail of the GAK. Participants were asked to fill out the questionnaire and return it using the enclosed envelope. By mid January 2002 a reminder letter was sent to non responders, and the 4^th ^of February 2002 was set as the latest return date. Completed and returned questionnaires were coded and entered in the SPSS software program and data were cleaned and made ready for statistical analysis.

### The questionnaire

The Maastricht Upper Extremity Questionnaire (MUEQ) Appendix 1 [see Additional file 1] assesses the occurrence and nature of CANS in computer workers and its associated physical and psychosocial risk factors. The MUEQ consists of 95 questions and has a completion time of approximately 20 minutes. The questionnaire covers the socio-demographic characteristics (age, gender, and employment status), as well as six main domains: i.e. (1) work station; (2) posture during work; (3) quality of break time; (4) job demands; (5) job control, and (6) social support. In addition, a number of items assess the quality of the work environment and the frequency and nature of extremity complaints, in the neck, shoulder, upper and lower arm, elbow, hand and wrist. Further, several items specified the clinical manifestations of the complaint: i.e. tingling, numbness, weakness, swelling, stiffness, fatigue, continuous pain and change in skin colour or temperature. The questions included in the MUEQ were partly derived from already existing instruments and partially developed by the authors themselves based on comprehensive study of the scientific and also grey literature. The items referring to potential psychosocial risk factors were based on the Job Content Questionnaire (JCQ) by Karasek et al [12]. The JCQ is a questionnaire-based instrument designed to measure the "content" of a respondent's work task(s) in a general manner. The questionnaire scales have been used to predict job related stress and coronary heart disease [12]. The MUEQ measures the key components of the job demand-control-support model by Karasek, namely skill discretion, decision authority, job demand and social support from supervisor and co-workers. Several items on physical work related risk factors were based on the Dutch Musculoskeletal Questionnaire (DMQ) by Hildebrandt et al [10]. As the present study is interested in measuring both the physical as well as the psychological workplace risk factors and because the psychometric properties of the JCQ and the DMQ independently have already been fairly well-established in other working populations [18,19]. Only items related to the presence of CANS were selected and combined in order to be included in the MUEQ.

The "complaint cases" were identified as participants who had complaints in at least one upper extremity body region for a minimal duration of one week during the preceding 12 months. All items were rephrased as statements in either a five point scale (completely true-completely false) and (always-never) or a dichotomous statement (yes-no). A simple English translation is presented in Appendix 1 [see Additional file 1] and the complete Dutch questionnaire is presented in Appendix 2 [see Additional file 1]. The English translation, as presented in Appendix 1, has not gone through a formal cross-cultural adaptation.

### Calculation of CANS prevalence rate

All statistical analyses were performed with the SPSS 12.0 software package. Prevalence rates of complaints over the past twelve months that lasted for at least one week were computed for each upper musculoskeletal body region (neck, shoulder, arm, elbow, hand and wrist) including 95% confidence intervals (CI).

### Factor analysis

Exploratory factor analysis was chosen as an appropriate approach since we aimed to analyse interrelations among a large number of items (questions) while trying to explain these variables in terms of their common underlying dimensions within each of the six domains [13,20]. The responses on various questions were conducted using Principal Component Analysis (PCA) with Varimax rotation. The number of factors retained was derived by considering the magnitude of the eigenvalues, Kaiser's (1960) eigenvalues [greater than 1] rule, the proportion of variance extracted, item content, and the interpretability of the resulting factors. Independent factors were considered as meaningful when they appeared before the break in the Scree plot results. As for factor loading after the Varimax rotation, items with a factor loading less then 0.5 on all factors were excluded, unless they represent an essential assumption. Further, each factor had to be comprised of at least three items. If the results indicated more than two factors then a forced two factor analysis was performed [13].

### Reliability and internal consistency of the questionnaire

The factor analysis assisted in exploring the different underlying factors (i.e. scales) within the different domains covered by the MUEQ. These scales may represent risk factors for the development of CANS.

One way to investigate the reliability of each scale is by calculating measures which indicate the internal consistency (i.e. homogeneity) of the items that form the scale. We investigated the internal consistency by calculating Cronbach's α for each scale and by calculating item-total correlations. Cronbach's α is a measure of how well each individual item in a scale correlates with the sum of the remaining items. An alpha greater than 0.70 was considered acceptable, this indicated that the individual items are independent but belong to the same scale. Optimal item-total correlation was considered to be between 0.2 and 0.5. Items with scores falling out of this range were examined for possible exclusion from the MUEQ [21].

### Performance of cross-validation

In order to test the stability of the factor structure cross-validation was carried out. This means that the results of both factor analysis and internal consistency analysis of one part of the data set are tested against the total data set, to ascertain that the two parts are as comparable as possible. The sub-sample was randomly selected from the study population and the comparison was made.

## Results

### Demographic characteristics of the study population

Two hundred and sixty four men and women out of the 600 responded to the baseline questionnaire which resulted in a response rate of 44% (n = 264). Questionnaires were filled out by an almost equal numbers of men (n = 133) and women (n = 131). The percentage of respondents working five days per week was higher among men (90%) than women (60%). Nevertheless, 55% of the women worked 6 to 8 hours per day with a computer in comparison to 44% of the men (Table 1).

**Table 1:** Descriptive characteristics of the study population (n = 264)

	Male (n = 133)	Female (n = 131)
Gender	50.4%	49.6%

Number of days working/Week		
3	0.8%	15.3%
4	8.3%	21.4%
5	90.2%	59.5%

Working 8 hours/Day	85.0%	66.4%

Working 6 to 8 hours with computer/Day	44.4%	55.7%

Number of working years in current position		
< 1	20.3%	23.7%
1–5	41.4%	43.5%
5–10	18.8%	14.5%
10–15	9.0%	8.4%
> 15	10.5%	9.2%

The data screening procedure showed no out of range cases. Moreover, all percentages of missing values were under 2% and scattered, and no systematic pattern was identified. Hence, the missing values were ignored. To examine whether the scores on the six main domains had a normal distribution, skewness and kurtosis of the domains were examined. The results (data not shown) indicated that there was no substantial deviation from normality in any of the main domains.

### Prevalence rates of CANS

Prevalence rates of CANS during the previous year that lasted at least one week are presented in table 2. The one-year prevalence rate of CANS indicated that 54% of the respondents reported at least one complaint in the arm, neck and shoulder of which 48% were male and 61% females. The most commonly reported complaints were neck and shoulder symptoms (33% and 31% respectively), followed by upper arm complaints and hand (12% and 11%), lower arm, wrist and elbow complaints (8%, 8% and 6%).

**Table 2:** Prevalence rates of upper extremity musculoskeletal complaints during the previous year that lasted at least one week

Localization of complaints	Total Number of subjects with complaints	Total Prevalence (95% CI) (n = 264)	Male Prevalence (95% CI) (n = 133)	Female Prevalence (95% CI) (n = 131)
Neck complaints	89	0.33 (0.27 to 0.39)	0.24 (0.17 to 0.31)	0.42 (0.33 to 0.50)
Shoulder complaints	81	0.31 (028 to 0.37)	0.20 (0.13 to 0.27)	0.42 (0.33 to 0.50)
Upper arm complaints	32	0.12 (0.08 to 0.16)	0.10 (0.05 to 0.15)	0.13 (0.07 to 0.18)
Elbow complaints	16	0.06 (0.03 to 0.08)	0.07 (0.03 to 0.11)	0.04 (0.01 to 0.07)
Lower arm complaints	21	0.08 (0.05 to 0.11)	0.06 (0.02 to 0.10)	0.09 (0.04 to 0.14)
Wrist complaints	21	0.08 (0.05 to 0.11)	0.06 (0.02 to 0.10)	0.09 (0.04 to0.14)
Hand complaints	30	0.11 (0.07 to 0.15)	0.10 (0.05 to 0.15)	0.12 (0.06 to 0.17)
Any upper extremity complaint	154	0.55(0.48 to 061)	0.48. (0.06 to 0.39)	0.61.(0.52 to 0.69)

The distribution of complaint by anatomical locality (i.e. left side, right side and both sides) classified by gender is presented in table 3 The results indicated that "right side" complaints are more frequently reported than "left side" complaints or "both sides" complaints. Exceptions were shoulder complaints at "both sides" which were more frequently reported than complaints localized on the right or left side (Table 3).

**Table 3:** Percentages of upper extremity musculoskeletal complaints distributed by anatomical area

**Percentages %**	**Localization of complaints**					
	
**Male (n = 133)**	Shoulder complaints	Upper arm complaints	Elbow complaints	Lower arm complaints	Wrist complaints	Hand complaints
Left Side	4.5	3.8	3.0	2.3	0.8	0.8
Right Side	5.3	4.5	3.6	3.0	3.8	3.8
Both Sides	10.5	2.3	0.8	0.8	1.5	6.0
**Female (n = 131)**						
Left Side	12.2	5.3	0.8	3.1	2.3	1.5
Right Side	7.6	6.1	3.1	3.8	2.3	4.6
Both Sides	22.9	2.3	0.8	2.3	5.3	6.1

**Figure 1:**
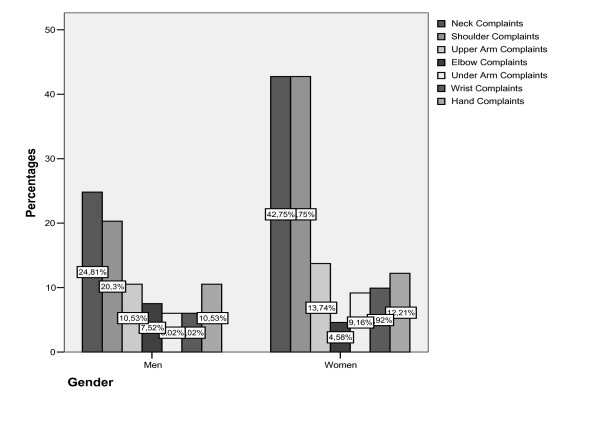
Percentages of upper extremity musculoskeletal complaints with a minimal duration of one week over the preceding year in groups according to gender.

Further, females reported higher prevalence rates for the various upper extremity anatomical areas (figure 1). The difference in the prevalence rates for neck and shoulder complaints between males and females were statistically significant (Table 2).

### Results of the cross-validation

We found that the number of factors, the factor structure and factors loadings were for the greater part comparable between the first randomly created sub-sample and the total sample. Differences were found for two items within the social support domain. Item "I find support from my supervisors" loads higher on the (social support) sub-scale in the first random sample. However, the item loads higher on the (work flow) sub-scale in the total sample. Further, the item "My work tasks depend on other colleagues" loads higher on the (work flow) sub-scale in the first sample but loads high on the (social support) sub-scale in the total sample.

Since no important differences were found in the results we only present the results of the factor analyses as applied to the randomly created sub sample. Table 7 percentages of participants' response to each item for the six main domains

**Table 7:** Percentage of response option for each item listed in the main domains

Items	Percentage % of response N = 264				
Work Station	No	Yes			
Desk at work has suitable height	14.8	85.2			
I can adjust my chair height	6.1	93.9			
When I use the mouse my arm is supported	33.4	65.9			
I have enough space to work at my desk	34.1	65.9			
Keyboard is placed directly in front of me	18.6	81.4			
Screen is placed directly in front of me	20.6	79.9			

Body Posture	Always	Often	Sometime	Seldom	Never
I sit for long hours in one position	40.1	29.0	16.0	7.6	7.6
I sit with lifted shoulders.	17.0	18.6	19.7	16.0	27.7
Sitting in awkward posture	7.6	21.2	29.9	23.1	18.2
Performing repetitive tasks	15.5	29.2	23.1	19.3	12.8
Job physically exhausting	2.3	36.1	18.2	35.2	8.3
Head is twisted towards the left or right	15.5	24.6	24	22.0	13.6
Trunk is twisted towards the left or right	3.4	13.3	16.7	32.6	34.0
I sit in asymmetrical position	5.3	15.2	16.7	32.6	34.1

Job Control					
I decide how to perform my job task	25.0	37.5	26.1	8.0	3.4
I participate with others in decision	17.4	30.3	31.3	14.4	6.4
I decide my own task changes	23.9	46.9	21.2	8.0	6.1
I determine the time & speed job tasks	26.9	37.9	13.0	3.0	1.9
I solve work problems by my self	23.9	40.9	21.2	8.0	6.1
My work develop my abilities	26.9	37.9	24.6	7.6	3.0
At work I learn new things	44.7	37.3	13.0	3.0	1.9
Creative in my work	30.3	30.7	25.8	8.7	4.5
I undertake different tasks in my work	22.3	34.1	32.2	7.6	3.8

Job Demand					
Work under extensive pressure	12.9	34.1	40.5	10.2	2.3
Difficulty to finish my job tasks	4.9	15.5	31.4	32.2	15.9
I take extra hours to finish	2.7	8.3	22.7	28.8	37.5
I speed to finish my tasks	6.1	21.2	42.4	22.7	17.0
No enough time to finish my job	2.3	12.1	33.3	35.2	17.0
I find my work tasks difficult	1.1	6.8	34.1	41.1	16.7
I have too many job tasks	2.3	12.1	33.3	35.2	17.0

Social Support	Always	Often	Sometime	Seldom	Never
My work sphere is good	1.9	6.8	23.9	41.3	26.1
I find support from colleges	4.6	8.3	22.3	33.3	31.3
I find support from supervisors	8.0	16.3	30.7	28.0	17.0
My colleagues are friendly	0.4	1.9	16.7	40.9	40.2
My supervisors are friendly	2.3	4.5	20.1	39.8	33.3
Work tasks depend on other colleges	6.8	14.8	33.7	33.7	11.0
The work flow goes smoothly	10.2	18.2	34.1	28.4	9.1
I can ask & enquire in my work	4.5	10.2	21.2	38.6	25.4

Break Time					
I can plan my work breaks	26.1	35.2	17.0	13.6	8.1
I can divide my work time	41.3	34.8	21.1	3.8	8.0
I can decide when to take a break	29.1	37.5	19.3	9.5	3.8
I alternate in my body posture	6.3	26.5	30.7	28.4	6.1
I alternate in my job task	11.5	29.2	37.7	17.8	6.8
I perform job task without computer	21.6	39.4	22.3	13.3	3.1
After two hours I take a break for 10 min	5.3	11.0	18.6	22.0	40.2

### Psychometric characteristics of the MUEQ

#### Work station

The first group of items addressed the work station domain (i.e. table, chair and computer position) and consisted of seven items. Three factors were extracted (data not shown) therefore, we undertook a forced two-factor solution. The rotated factor loadings of these analyses are shown in table 4. Examination of the factor loadings showed that one item "My chair support my lower back" load poorly on both factors and therefore was deleted.

**Table 4:** Factor loadings identified using principal component analysis and the orthogonal VARIMAX rotation *

Domain	Abbreviated item description	Factor 1	Factor 2
Work Station		Office equipment	Computer position
	Desk at work has suitable height	0.80	-0.06
	I can adjust my chair height	0.62	0.16
	When I use the mouse my arm is supported	0.50	0.16
	I have enough space to work at my desk	0.66	0.16
	Keyboard is placed directly in front of me	0.14	0.87
	Screen is placed directly in front of me	0.05	0.77
Eigenvalue		1.84	1.28
% of Variance		26.1	18.3

Body Posture		Awkward body posture	Head and body posture
	I sit for long hours in one position	0.59	0.12
	I sit with lifted shoulders.	0.72	0.10
	Sitting in awkward posture	0.71	0.27
	Performing repetitive tasks	0.77	0.02
	Job physically exhausting	0.50	0.06
	Head is twisted towards the left or right	0.30	0.74
	Trunk is twisted towards the left or right	0.08	0.82
	I sit in asymmetrical position	0.26	0.61
Eigenvalue		3.30	1.45
% of Variance		30.0	13.9

Job Control		Decision authority	Skill discretion
	I decide how to perform my job task	0.74	0.04
	I participate with others in decision	0.71	0.32
	I decide my own task changes	0.68	0.39
	I determine the time & speed job tasks	0.55	0.12
	I solve work problems by my self	0.74	0.04
	My work develop my abilities	0.28	0.62
	At work I learn new things	0.05	0.68
	Creative in my work	0.16	0.66
	I undertake different tasks in my work	0.05	0.67
Eigenvalue		3.32	1.33
% of Variance		36.9	14.4

Job Demands		Work pressure	Task complexity
	Work under extensive pressure	0.76	0.10
	Difficulty to finish my job tasks	0.83	0.24
	I take extra hours to finish	0.70	0.11
	I speed to finish my tasks	0.59	0.26
	No enough time to finish my job	0.73	0.02
	I find my work tasks difficult	0.06	0.92
	I have too many job tasks	0.23	0.89
Eigenvalue		3.225	1.36
% of Variance		46.5	19.5

Social Support		Social support	Work flow
	My work sphere is good	0.69	0.14
	I find support from colleges	0.65	0.56
	I find support from supervisors	0.84	0.16
	My colleagues are friendly	0.85	0.18
	My supervisors are friendly	0.63	0.47
	Work tasks depend on other colleges	0.51	0.62
	The work flow goes smoothly	0.08	0.76
	I can ask & enquire in my work	0.15	0.74
Eigenvalue		3.96	1.04
% of Variance		49.9	13.0

Break Time		Autonomy	Break quality
	I can plan my work breaks	0.82	0.11
	I can divide my work time	0.76	0.02
	I can decide when to take a break	0.76	0.12
	I alternate in my body posture	0.07	0.76
	I alternate in my job task	0.20	0.82
	I perform job task without computer	0.39	0.53
	After two hours I take a break for 10 min	0.03	0.57
Eigenvalue		2.06	2.06
% of Variance		35.5	25.9

* Only items with a factor loading >0.5 on at least one factor are reported

The first factor included four items related to the "office equipment". This sub scale had a low Cronbach's alpha of 0.51 which is below the accepted norm of 0.70 [21] and values of item-total correlations were between 0.14 to 0.32 (Table 5 and 6). Two items (keyboard is placed directly in front of me and screen is placed directly in front of me) load convincingly on the second factor. They were grouped as "computer position" and this accounted for 18.3% of the total variance. Cronbach's alpha was 0.75 and item-total correlation of the sub-scale was 0.59 (Table 5 and 6).

**Table 5:** Internal consistency of the fourteen subscales

Domain	Sub Scales	Internal consistency (Cronbach's α)	Items numbers
Work Station	Sub scale 1: Office equipment	.51	10.11.12.16
	Sub scale 2: Computer position	.75	14.15
Body Posture	Sub scale 3: Awkward body posture	.71	18.19.20.21.22
	Sub scale 4: Head and body posture	.54	25.26.27
Job Control	Sub scale 5: Decision authority	.76	28.29.30.31.32
	Sub scale 6: Skill discretion	.69	33.34.35.36
Job Demand	Sub scale 7: Work pressure	.80	37.38.39.40.41
	Sub scale 8: Task complexity	.84	42.43
Social Support	Sub scale 9: Social support	.80	64.65.66.67.68
	Sub scale 10: Work flow	.60	61.62.63
Break Time	Sub scale 11: Autonomy	.71	44.45.46
	Sub scale 12: Break quality	.70	47.48.49.50

**Table 6:** Item-total correlation of the fourteen sub scales

Domain	Sub Scales	Item-total correlation (Min-Max)	Items numbers
Work Station	Sub scale 1: Office equipment	0.14 to 0.32	10.11.1216
	Sub scale 2: Computer position	0.59	14.15
Body Posture	Sub scale 3: Awkward body posture	0.30 to 0.57	18.19.20.21.22
	Sub scale 4: Head and body posture	0.38 to 0.45	25.26.27
Job Control	Sub scale 5: Decision authority	0.37 to 0.61	28.29.30.31.32
	Sub scale 6: Skill discretion	0.28 to 0.51	33.34.35.36
Job Demand	Sub scale 7: Work pressure	0.50 to 0.75	37.38.39.40.41
	Sub scale 8: Task complexity	0.54	42.43
Social Support	Sub scale 9: Social support	0.58 to 0.67	64.65.66.67.68
	Sub scale 10: Work flow	0.28 to 0.38	61.62.63
Break Time	Sub scale 11: Autonomy	0.47 to 0.58	44.45.46
	Sub scale 12: Break quality	0.39 to 0.60	47.48.49.50

#### Body posture

The second domain addressed body posture and consisted of eleven items (Appendix 1). Two factors were extracted and examination of the rotated factor loadings showed that three items (when I key my hand is placed in a straight line with my lower arm, head is bent, and I keep a good work posture.) load poorly (<0.5) on both factors. They were therefore excluded. Five items related to "awkward body posture" load highly on the first factor and this accounted for 30.0% of the total variance (Table 4). The scale had an acceptable Cronbach's alpha of 0.71 and item-total correlation of this scale ranged from 0.30 to 0.57 (Table 5 and 6). Furthermore, three items related to "head and body posture" load highly on the second factor and this accounted for 13.9% of the total variance. The scale had a low Cronbach's alpha of 0.54 and Item-total correlation of this scale ranged from 0.38 to 0.45.

#### Job control

The job control domain included nine items. The Scree plot identified two factors; the rotated factor loadings indicated that the first factor "decision authority" contained five items (Table 4). This accounted for 36.9% of the total variance and Cronbach's alpha was 0.76 and the Item-total correlation ranged from 0.37 to 0.61 (Table 5 and 6). The second factor "skill discretion" contained four items (Table 4). These accounted for 14.4% of the total variance with Cronbach's alpha of 0.69, which is below the accepted norm of 0.70 [21] and Item-total correlations ranging from 0.28 to 0.51 (Table 5 and 6).

#### Job demands

The job demands domain consisted of a total number of seven items (Appendix 1). The Scree plot applied to the data shows that two factors were to be retained. Examination of the rotated factor loading showed that two items (I find my work tasks difficult and I have too many job tasks) load high on the second factor. Although the factor holds less than three items they nevertheless covered an important assumption of worker's perception of job pressure and were therefore retained and labelled "task complexity". These factors accounted for 19.5% of the total variance (Table 4). Cronbach's alpha was 0.84 and the Item-total correlation was 0.54. The other five items (Table 4) constituted the "work pressure" scale which accounted for 46.5% of the total variance. Cronbach's alpha was 0.80 and the Item-total correlations ranged from 0.50 to 0.75 (Table 5 and 6).

#### Social support

Eight items investigated the relationship among co-workers and between workers and supervisors. The Scree plot indicated that two factors were meaningful enough to be retained. The rotated factor loadings indicated that five items load high on the first factor "social support" and this accounted for 49.9% of the total variance (Table 4). Cronbach's alpha had an acceptable level of 0.80 and item-total correlations of "social support" ranged from 0.58 to 0.67. The other three items (the work flow goes smoothly, I can ask and enquire in my work, and work tasks depend on other colleges) were classified as the "work flow" and accounted for 13.0 % of the total variance. Cronbach's was 0.60 and item-total correlations ranged from 0.28 to 0.38 (Table 5 and 6).

#### Break time

Break time during working hours was investigated by eight items. The rotated factor loading, indicated that, one item (I find my work breaks sufficient) loads poorly on both factors and was deleted. The first factor holds three items which made the "autonomy" scale and this accounted for 35.5% of the total variance (Table 4). Cronbach's alpha was 0.71 and the item-total correlations of the autonomy scale ranged from 0.47 to 0.58. Four items related to the "break quality" load highly on the second factor, accounting for 25.9% of the total variance, Cronbach's alpha of 0.70 and with item-total correlations ranging from 0.39 to 0.60 (Table 5 and 6).

## Discussion

In the present sample of 264 subjects of computer office workers, we assessed the 12-months prevalence of CANS among a Dutch cohort of computer workers and tested a newly developed comprehensive questionnaire (MUEQ) that included various scales, which are hypothetically related to an increased risk for CANS. The results showed higher prevalence of neck and shoulder complaints in the study population compared to arm, elbow and hand complaints. This matter is rather debatable in the literature since some reviews, which focus on muscle activity during computer use, suggest a stronger relationship between computer use and complaints of the hand and arm, than to complaints of the neck and shoulders [22,23]. On the other hand, there are also studies indicating that neck or shoulder complaints are more common than other complaints in the upper extremity in computer workers [1,9,22]. Job characteristics, such as high quantitative job demands, having little influence on one's work situation, and limited rest break opportunities have been found as predictors of neck pain [24, 25]. One should cautiously postulate that certain risk factors are more related to the presence of neck and shoulder pain than to wrist and hand pain. However, the majority of the available literature either investigates a variety of symptoms (frequency, intensity) and/or one body region or the used concepts are rather broadly defined which complicates making a meaningful comparison of the results presented in the literature [25]. However, the correlations between risk factor scales and CANS are to be investigated and presented in a separate manuscript.

Furthermore, musculoskeletal complaints and neck and shoulder complaints in particular, were more common among women than among men. Even though women and men were working in the same institute and had the same job titles. Another study [26] confirmed our findings, showing the prevalence of symptoms in the neck and upper extremities per year ??to be 51% among men and 72% among women. A possible explanation for this gender related difference is that women apparently do not always perform tasks with the same physical requirements or work organization as men. The European Foundation for the Improvement of Living and Working Conditions indicated that women tended to perform more repetitive work on average, whereas men were less likely to sit for prolonged periods compared to women (refer). Furthermore, women are more often exposed to additional stress from unpaid work such as housekeeping and child care [18, 26].

We have attempted to accurately examine the measurement properties of the MUEQ, but there are some aspects and limitations that merit discussion. The response rate of 44% was rather low. It is possible that a 95-item survey was too long for routine administration among workers. Hence, our results must be considered in light of the limitations associated with the study response rate.

The results of the factor analysis in the present study indicated that each domain included two scales accounting for approximately 50% of the variance. Identification of the domains can be gleaned from the pattern of the item loadings on the two scales. The factor loading was not difficult to interpret as there was a semantic relationship between the items that contribute to it. For instance, the job control domain was subdivided into a decision authority scale, which reflects worker's perception of their control in performing job task, and a skill discretion scale, which addresses information about skills needed to perform job tasks. The overlap between these two concepts was in line with Karasek's (1989) results which indicated that these two concepts are often combined as decision latitude [20]. However, the results of the present study gave reason to treat these control concepts as separate, yet related, concepts.

As for the reliability coefficient, Nunnally (1978) suggested a coefficient of at least 0.70 to be acceptable [13]. Cronbach's alpha coefficients for the majority of the sub-scales in the MUEQ were larger than 0.70. Some of the items in the social support and work station scales showed sub optimal item-total correlation (either below 0.2 or above 0.5). Cronbach's alpha did not improve sufficiently when items were removed to justify the omission of items from the scale.

The study abstained from calculating the internal consistency with regard to the quality of work environment domain because the meaning of the items included was too different in order to be clustered in one scale.

## Conclusion

In conclusion, neck and shoulder complaints are reported more frequently than complaints in any of the other upper body regions. Further, women had higher 12-months prevalence rates of upper extremity musculoskeletal complaints than men. The tested questionnaire, i.e. the MUEQ, has satisfactory reliability and internal consistency when used to document CANS among computer office workers in the Netherlands.

This questionnaire can be used as a screening inventory for occupational health services to identify worker groups in which a more thorough ergonomic analysis is indicated.

We tested the psychometric properties of the questionnaire in employees without severe musculoskeletal complaints. Further evaluation of the psychometric properties of the questionnaire studies in other populations may therefore be useful. The question whether the scales identified by the factor analyses are indeed risk factors for the development of CANS in computer workers needs to be addressed in well designed prospective cohort studies.

## Competing interests

The author(s) declare that they have no competing interests.

## Authors' contributions

S.E and J.B.S have made substantial contributions to conception and writing. S E did the data analysis and drafted the manuscript. J.K, P.H.G. L and R Bhave helped in designing the study questionnaire and critically revised the manuscript. All authors read and approved the final manuscript.

## Pre-publication history

The pre-publication history for this paper can be accessed here:



## Supplementary Material

Additional file 1 Appendix 1 and 2 Maastricht Upper Extremity Questionnaire (MUEQ). Appendix 1 presents the English translated version of the MUEQ and Appendix 2 presents the original Dutch version. Click here for file
