# Supplementary material for: Prevalence of complaints of arm, neck and shoulder among computer office workers and psychometric evaluation of a risk factor questionnaire
Source: BMC Musculoskelet Disord. 2007 Jul 14;8:68. doi: 10.1186/1471-2474-8-68 (PMC1952062; doi:10.1186/1471-2474-8-68)
Supplement: Additional file 1 — Appendix 1 and 2 Maastricht Upper Extremity Questionnaire (MUEQ). Appendix 1 presents the English translated version of the MUEQ and Appendix 2 presents the original Dutch version. [file 1471-2474-8-68-S1.doc]

# Appendix 1

**Maastricht Upper Extremity Questionnaire (MUEQ)**

**General Information:**

|  | Gender | □ Male □ Female |
| --- | --- | --- |
|  | Surname | ____________________________________ |
|  | Date of birth? | ___-___-19___ |
|  | Where do you work? | □ Heerlen  □ Maastricht  □ Both |
|  | What is your current position? | ____________________________________ |
|  | How long have you been working in this position? | ___ Year |
|  | How many days do you work per week?  (*over time not included*) | ___ Day |
|  | How many hours do you work per day?  *(breaks and over time not included)* | ___ Hour |
|  | How many hours per working day do you work behind your computer? | ___ Hour |

**Work Station**

|  | My desk (table) at work has suitable height. | □ No  □ Yes |
| --- | --- | --- |
|  | I can adjust my chair height. | □ No  □ Yes |
|  | When I use the mouse device, my arm is supported by the table. | □ No  □ Yes |
|  | The chair I use during work supports my lower back. | □ No  □ Yes |
|  | My keyboard is placed directly in front of me. | □ No  □ Yes |
|  | The screen is placed directly in front of me. | □ No  □ Yes |
|  | I have enough space to work at my office. | □ No  □ Yes |

**Body Posture**

|  | |  | | Always | | Often | | Someti-mes | | Seldom | | Never |
| --- | --- | --- | --- | --- | --- | --- | --- | --- | --- | --- | --- | --- |
|  | | During my work I keep a good work posture. | | □ | | □ | | □ | | □ | | □ |
|  | | At work I sit for long hours in one position. | | □ | | □ | | □ | | □ | | □ |
|  | | For more than two hours per day I sit with lifted shoulders. | | □ | | □ | | □ | | □ | | □ |
|  | | During my work I sit in awkward posture. | | □ | | □ | | □ | | □ | | □ |
|  | In work I perform repetitive tasks. | | □ | | □ | | □ | | □ | | □ | |
|  | I find my job physically exhausting. | | □ | | □ | | □ | | □ | | □ | |
|  | When I key my hand is placed in a straight line with my lower arm. | | □ | | □ | | □ | | □ | | □ | |
|  | When I work my head is bended. | | □ | | □ | | □ | | □ | | □ | |
|  | Head is twisted towards the left or right. | | □ | | □ | | □ | | □ | | □ | |
|  | Trunk is twisted towards the left or right. | | □ | | □ | | □ | | □ | | □ | |
|  | My Trunk is in asymmetrical position. | | □ | | □ | | □ | | □ | | □ | |

Job Control

|  |  | Always | Often | Someti-mes | Seldom | Never |
| --- | --- | --- | --- | --- | --- | --- |
|  | I decide how to perform my job task. | □ | □ | □ | □ | □ |
|  | I participate with others in decision taking. | □ | □ | □ | □ | □ |
|  | I decide my own task changes. | □ | □ | □ | □ | □ |
|  | I determine the time & speed job tasks. | □ | □ | □ | □ | □ |
|  | I solve work problems by my self. | □ | □ | □ | □ | □ |
|  | My work develops my abilities. | □ | □ | □ | □ | □ |
|  | In my work I learn new things. | □ | □ | □ | □ | □ |
|  | I have to be creative in my work. | □ | □ | □ | □ | □ |
|  | I under take different tasks in my work. | □ | □ | □ | □ | □ |

Job Demand

|  |  | Always | Often | Someti-mes | Seldom | Never |
| --- | --- | --- | --- | --- | --- | --- |
|  | I work under extensive work pressure. | □ | □ | □ | □ | □ |
|  | I find it difficult to finish my tasks on time. | □ | □ | □ | □ | □ |
|  | I take extra hours to finish my job tasks. | □ | □ | □ | □ | □ |
|  | I have no enough time to finish my job task. | □ | □ | □ | □ | □ |
|  | At work I speed to finish my tasks on time. | □ | □ | □ | □ | □ |
|  | I find my work tasks difficult. | □ | □ | □ | □ | □ |
|  | I have too many job tasks. | □ | □ | □ | □ | □ |

Break Time

|  |  | Always | Often | Someti-mes | Seldom | Never |
| --- | --- | --- | --- | --- | --- | --- |
|  | I can plan my work breaks. | □ | □ | □ | □ | □ |
|  | I can divide my work time. | □ | □ | □ | □ | □ |
|  | I can decide when to take a break. | □ | □ | □ | □ | □ |
|  | I alternate in my body posture. | □ | □ | □ | □ | □ |
|  | I alternate in my job task. | □ | □ | □ | □ | □ |
|  | I perform job task without computer. | □ | □ | □ | □ | □ |
|  | After two hours I take a break for 10 minutes. | □ | □ | □ | □ | □ |
|  | I find my work breaks sufficient. | □ | □ | □ | □ | □ |

Work environment

|  |  | Always | Often | Someti-mes | Seldom | Never |
| --- | --- | --- | --- | --- | --- | --- |
|  | I find my work environment good. | □ | □ | □ | □ | □ |
|  | The air inside the office is too dry. | □ | □ | □ | □ | □ |
|  | The air inside the office is too cold. | □ | □ | □ | □ | □ |
|  | In the office there is unwanted air. | □ | □ | □ | □ | □ |
|  | There is available fresh air in my work. | □ | □ | □ | □ | □ |
|  | My work environment is noisy. | □ | □ | □ | □ | □ |
|  | My work place is too bright. | □ | □ | □ | □ | □ |
|  | I gaze at the computer screen. | □ | □ | □ | □ | □ |
|  | The computer screen reflects the office lights. | □ | □ | □ | □ | □ |

Social Support

|  |  | Always | Often | Someti-mes | Seldom | Never |
| --- | --- | --- | --- | --- | --- | --- |
|  | The work flow goes smoothly. | □ | □ | □ | □ | □ |
|  | I can ask and enquire in my work. | □ | □ | □ | □ | □ |
|  | My work tasks depend on other colleges. | □ | □ | □ | □ | □ |
|  | My work atmosphere is comfortable. | □ | □ | □ | □ | □ |
|  | If I made a mistake in my work task I find support from my colleges. | □ | □ | □ | □ | □ |
|  | If I made a mistake in my work task I find support from my supervisors. | □ | □ | □ | □ | □ |
|  | My colleagues are friendly. | □ | □ | □ | □ | □ |
|  | My supervisors are friendly. | □ | □ | □ | □ | □ |

Complaints

During the past year I had pain or complaints for at least one week in one or more of the following body regions

|  | Neck | □ No  □ Yes |  |  |
| --- | --- | --- | --- | --- |
|  | Shoulder(s) | □ No  □ Yes → | If Yes, | □ Left  □ Right  □ Both |
|  | Upper Arm | □ No  □ Yes → | If Yes, | □ Left  □ Right  □ Both |
|  | Elbow (s) | □ No  □ Yes → | If Yes, | □ Left  □ Right  □ Both |
|  | Lower Arm | □ No  □ Yes → | If Yes, | □ Left  □ Right  □ Both |
|  | Wrists | □ No  □ Yes → | If Yes, | □ Left  □ Right  □ Both |
|  | Hand | □ No  □ Yes → | If Yes, | □ Left  □ Right  □ Both |

From here (upper musculoskeletal extremity) would be used to represent (neck, shoulder, hand, wrist, arm and elbow)

|  | During the past year I had pain/complaint/disability in my upper musculoskeletal extremity | □ No  □ Yes |
| --- | --- | --- |
|  | The longest period of complaint (in the past year) whereby I could not perform my daily activity was | □ ____Days  □ ____weeks |
|  | During the past year I was referred to the physician due to my upper extremity pain? | □ No  □ Yes→ The physician. Diagnosis of the complaint was? _________ |
|  | What kind of treatment did you receive (during the past year) | □ Physiotherapy  □ Medication  □ Operation  □ Other ______________ |
|  | Because of my upper extremity pain I have lost a job before | □ No  □ Yes |
|  | Because of my upper extremity complaints (during the past year) I was absent from work | □ No  □ Yes |
|  | Due to upper extremity complaints in the past year my activities were hindered  - in my work  - in my leisure time | □ No  □ Yes  □ No  □ Yes |
|  | My complaints are due to a previous accident. | □ No  □ Yes |

**The next questions are related to pain complaints in the neck, shoulder, hand, wrist, and elbow in the past year**

|  | I feel pain in my upper musculoskeletal extremity as soon as I finish work | □ No  □ Yes → | This pain disappears after a short rest | □ No  □ Yes |
| --- | --- | --- | --- | --- |
|  | I feel fatigue and exhaustion in my upper musculoskeletal extremity | □ No  □ Yes → | This complaint disappears after a short rest | □ No  □ Yes |
|  | I feel stiffness in my finger | □ No  □ Yes → | This stiffness disappears after a short rest | □ No  □ Yes |
|  | I feel numbness in my fingers | □ No  □ Yes → | This numbness continues after a short rest | □ No  □ Yes |
|  | I feel tingling in my fingers | □ No  □ Yes → | This tingling continue after work | □ No  □ Yes |
|  | I feel weakness in my upper musculoskeletal extremity | □ No  □ Yes → | This weakness continue after work | □ No  □ Yes |
|  | I suffer from swelling in my hands | □ No  □ Yes → | This swelling continue after work | □ No  □ Yes |
|  | I feel swelling/ stiffness in my upper musculoskeletal extremity | □ No  □ Yes |  |  |
|  | I feel continuous pain in my upper musculoskeletal extremity | □ No  □ Yes |  |  |
|  | I feel a change in the colour, temperature, sweating in my upper musculoskeletal extremity | □ No  □ Yes |  |  |
|  | I use mouse pad, file holder, foot supporter to reduce upper musculoskeletal extremity pain | □ No  □ Yes |  |  |
|  | I use neck collar or belts or other to reduce upper musculoskeletal extremity pain | □ No  □ Yes |  |  |

**Appendix 2**

**The original Dutch scale**

Algemeen

|  | Wat is uw geslacht? | □ man □ vrouw |
| --- | --- | --- |
|  | Wat is uw achternaam (meisjesnaam) + voorletters? | ________________________________________ |
|  | Wat is uw geboortedatum? | ___-___-19___ |
|  | Waar werkt u?  *naam bedrijf, vestigingsadres* | □ Heerlen  □ Maastricht  □ Beide |
|  | Wat is uw huidige functie? | ________________________________________ |
|  | Hoeveel jaar bekleedt u reeds die functie? | ___ jaar |
|  | Hoeveel dagen werkt u gemiddeld per week? Overwerk niet meegerekend | ___ dagen |
|  | Hoeveel uren werkt u gemiddeld per dag? Pauzes en overwerk niet meegerekend | ___ uren |
|  | Hoeveel uren werkt u gemiddeld per dag achter een beeldscherm? | ___ uren |

**Werk plek:**

|  | Mijn bureau heeft een voor mij geschikte werkhoogte | □ nee  □ ja |
| --- | --- | --- |
|  | Ik kan mijn bureaustoel in hoogte verstellen | □ nee  □ ja |
|  | Ik kan mijn stoel goed aanschuiven  *(met buik tegen tafel is mogelijk)* | □ nee  □ ja |
|  | Mijn arm kan ondersteund worden bij gebruik van de muis | □ nee  □ ja |
|  | Tijdens het werk geeft de stoel mij steun in de onderrug | □ nee  □ ja |
|  | Het toetsenbord staat recht voor mij | □ nee  □ ja |
|  | Ik kan recht voor het beeldscherm zitten | □ nee  □ ja |

**Houding**

|  | |  | | Altijd  1 | | 2 | | 3 | | 4 | | Nooit  5 |
| --- | --- | --- | --- | --- | --- | --- | --- | --- | --- | --- | --- | --- |
|  | | Ik vind dat ik werk in een goede werkhouding | | □ | | □ | | □ | | □ | | □ |
|  | | Tijdens mijn werk zit ik lang achtereen in dezelfde werkhouding *(bijv. zitten of staan)* | | □ | | □ | | □ | | □ | | □ |
|  | | Ik zit meer dan twee uur per dag met opgeheven schouders  *Het verschil tussen ontspannen en opgeheven schouders merkt u als u beide situaties uitprobeert.* | | □ | | □ | | □ | | □ | | □ |
|  | | Tijdens mijn werk zit ik vaak in een ongemakkelijke houding | | □ | | □ | | □ | | □ | | □ |
|  | Tijdens mijn werk moet ik voortdurend lang achtereen dezelfde bewegingen maken | | □ | | □ | | □ | | □ | | □ | |
|  | Ik vind mijn werk lichamelijk inspannend | | □ | | □ | | □ | | □ | | □ | |
|  | Als ik typ staan mijn handen in een rechte lijn met mijn onderarm  *Dit is het geval als u een rechte lijn kunt trekken van elleboog naar pols en middenvinger* | | □ | | □ | | □ | | □ | | □ | |
|  | Ik werk met mijn hoofd gebogen  *Bijv. als documenten tijdens het typen naast u liggen dus bij niet-blind typen.* | | □ | | □ | | □ | | □ | | □ | |
|  | Ik werk met mijn hoofd gedraaid *Bijv. als documenten naast u liggen tijdens typen* | | □ | | □ | | □ | | □ | | □ | |
|  | Ik zit met mijn romp gedraaid *Bijv. als u niet recht achter uw toetsenbord zit* | | □ | | □ | | □ | | □ | | □ | |
|  | Ik zit in een asymmetrische / kromme houding *Bijv. als u op 1 arm leunt tijdens muizen* | | □ | | □ | | □ | | □ | | □ | |

Werk Inhoud

|  |  | Altijd  1 | 2 | 3 | 4 | Nooit  5 |
| --- | --- | --- | --- | --- | --- | --- |
| 28 | In mijn werk kan ik zelf beslissen hoe ik mijn werk uitvoer | □ | □ | □ | □ | □ |
| 29 | Ik kan meebeslissen over wat er in mijn werk gebeurt | □ | □ | □ | □ | □ |
|  | Ik krijg in mijn werk de ruimte om beslissingen zelf te nemen | □ | □ | □ | □ | □ |
|  | Ik kan mijn werk zelf indelen en het werktempo zelf bepalen | □ | □ | □ | □ | □ |
|  | Ik krijg op mijn werk voldoende ruimte om zelf problemen op te lossen | □ | □ | □ | □ | □ |
|  | Mijn werk doet voldoende beroep op mijn vaardigheden en capaciteiten | □ | □ | □ | □ | □ |
|  | Mijn baan vereist dat ik nieuwe dingen leer | □ | □ | □ | □ | □ |
|  | In mijn werk moet ik creatief zijn | □ | □ | □ | □ | □ |
|  | Mijn baan vereist dat ik snel werk | □ | □ | □ | □ | □ |

**Werk Druk**

|  |  | Altijd  1 | 2 | 3 | 4 | Nooit  5 |
| --- | --- | --- | --- | --- | --- | --- |
|  | Ik vind dat ik onder een hoge werkdruk moet presteren | □ | □ | □ | □ | □ |
|  | Ik heb moeite mijn werk op tijd af te krijgen | □ | □ | □ | □ | □ |
|  | Ik moet regelmatig overwerken, omdat ik mijn werk niet op tijd kan afronden | □ | □ | □ | □ | □ |
|  | Ik heb geen genoeg tijd om mijn werk af te maken | □ | □ | □ | □ | □ |
|  | Tijdens mijn werk moet ik maximale snelheid werken | □ | □ | □ | □ | □ |
|  | Ik vind mijn werk zwaar | □ | □ | □ | □ | □ |
|  | Ik vind mijn werk moeilijk | □ | □ | □ | □ | □ |

Pauzes

|  |  | Altijd  1 | 2 | 3 | 4 | Nooit  5 |
| --- | --- | --- | --- | --- | --- | --- |
|  | Ik kan tijdens mijn werk zelf mijn pauzes inplannen | □ | □ | □ | □ | □ |
|  | Ik kan mijn werk zelf onderbreken als ik dat nodig vind | □ | □ | □ | □ | □ |
|  | Ik kan het tijdstip waarop ik begin en stop met werken zelf indelen | □ | □ | □ | □ | □ |
|  | Tijdens het werk wissel ik regelmatig van lichaamshouding | □ | □ | □ | □ | □ |
|  | Tijdens het werk wissel ik taken af | □ | □ | □ | □ | □ |
|  | Ik wissel mijn werk met de computer af met andere bezigheden | □ | □ | □ | □ | □ |
|  | Ik neem na maximaal 2 uur werken achter de computer een wat langere pauze van tenminste 10 minuten | □ | □ | □ | □ | □ |
|  | Op een werkdag heb ik voldoende pauzes | □ | □ | □ | □ | □ |

**Werk Omgeving**

|  |  | Altijd  1 | 2 | 3 | 4 | Nooit  5 |
| --- | --- | --- | --- | --- | --- | --- |
|  | Ik vind dat mijn werkomgeving goed is | □ | □ | □ | □ | □ |
|  | De lucht is vaak te droog | □ | □ | □ | □ | □ |
|  | Het is koud op de werkplek | □ | □ | □ | □ | □ |
|  | Er is sprake van een hinderlijke tocht op de werkplek | □ | □ | □ | □ | □ |
|  | Op mijn werk heb ik gebrek aan frisse lucht | □ | □ | □ | □ | □ |
|  | Het is rumoerig op de werkplek, waardoor ik gestoord wordt in mijn werk | □ | □ | □ | □ | □ |
|  | De werkplek is te licht | □ | □ | □ | □ | □ |
|  | Ik moet turen op het beeldscherm | □ | □ | □ | □ | □ |
|  | Er is sprake van een hinderlijke spiegeling in het scherm | □ | □ | □ | □ | □ |

**Sociale steun**

|  |  | Altijd  1 | 2 | 3 | 4 | Nooit  5 |
| --- | --- | --- | --- | --- | --- | --- |
|  | Het werk is doorgaans goed georganiseerd | □ | □ | □ | □ | □ |
|  | Ik kan voldoende overleggen over mijn werk | □ | □ | □ | □ | □ |
|  | Ik erger me vaak aan anderen op het werk | □ | □ | □ | □ | □ |
|  | Ik ervaar de werksfeer als prettig | □ | □ | □ | □ | □ |
|  | Als ik fouten maak op mijn werk is er voldoende hulp en steun van mijn collega's | □ | □ | □ | □ | □ |
|  | Als ik fouten maak op mijn werk is er voldoende hulp en steun van leidinggevenden | □ | □ | □ | □ | □ |
|  | Mijn collega's zijn vriendelijk | □ | □ | □ | □ | □ |
|  | Mijn leidinggevenden zijn vriendelijk | □ | □ | □ | □ | □ |

Klachten

Gedurende het afgelopen jaar had ik minimaal een week klachten/pijn aan:

|  | Nek | □ nee  □ ja |  |  |
| --- | --- | --- | --- | --- |
|  | Schouder(s) | □ nee  □ ja → | zo ja, | □ links  □ rechts  □ beide |
|  | Bovenarm(en) | □ nee  □ ja → | zo ja, | □ links  □ rechts  □ beide |
|  | Elleboog(en) | □ nee  □ ja → | zo ja, | □ links  □ rechts  □ beide |
|  | Onderarm(en) | □ nee  □ ja → | zo ja, | □ links  □ rechts  □ beide |
|  | Pols(en) | □ nee  □ ja → | zo ja, | □ links  □ rechts  □ beide |
|  | Hand(en) | □ nee  □ ja → | zo ja, | □ links  □ rechts  □ beide |

##### **Vanaf hier wordt de term 'bovenste extremiteit' gebruikt voor**

**nek, schoudergordel, arm, elleboog, pols en/of hand)**

|  | Ik heb ooit pijn of klachten gehad aan mijn bovenste extremiteit | □ nee  □ ja |
| --- | --- | --- |
|  | De totale duur van de periode waarin ik mijn normale dagelijkse activiteiten niet kon doen als gevolg van klachten is: | □ minder dan een week  □ meer dan een week, namelijk _____ weken |
|  | In de afgelopen 12 maanden gaven mijn lichamelijke klachten aanleiding om een arts te bezoeken. | □ nee  □ ja → heeft deze de diagnose_________ |
|  | Indien u een behandeling heeft ondergaan, welke?  *Meerdere antwoorden mogelijk* | □ fysiotherapie  □ medicatie  □ operatie  □ anders, nl. _____________________________ |
|  | Ik heb ooit ander werk moeten gaan doen in verband met dergelijke lichamelijke klachten. | □ nee  □ ja |
|  | Vanwege klachten aan mijn bovenste extremiteit heb ik gedurende de laatste 12 maanden het werk moeten verzuimen | □ nee  □ ja |
|  | Tijdens periodes van klachten ben ik in de laatste 12 maanden minder actief geweest :  - op mijn werk  - in mijn vrije tijd | □ nee  □ ja  □ nee  □ ja |
|  | Heeft u op dit moment nog klachten aan uw bovenste extremiteit, die het gevolg zijn van verwondingen na een ongeval | □ nee  □ ja |

**De nu volgende vragen gaan over uw laatst doorgemaakte klachtenperiode binnen een jaar**

|  | Nadat ik gewerkt heb, heb ik pijn in mijn bovenste extremiteit | □ nee  □ ja → | verdwijnen deze klachten na rust? | □ nee  □ ja |
| --- | --- | --- | --- | --- |
|  | Vermoeidheid in de bovenste extremiteit | □ nee  □ ja → | verdwijnen deze klachten na rust? | □ nee  □ ja |
|  | Kramp in mijn vingers | □ nee  □ ja → | verdwijnen deze klachten na rust? | □ nee  □ ja |
|  | Een doof gevoel in mijn vingers | □ nee  □ ja → | verdwijnen deze klachten na rust? | □ nee  □ ja |
|  | Irritatie en/of prikkelingen in de vingers | □ nee  □ ja → | blijft dit gevoel aanwezig in de uren na het werk? | □ nee  □ ja |
|  | Slapheid en/of krachtsverlies in mijn bovenste extremiteit | □ nee  □ ja → | blijft dit gevoel aanwezig in de uren na het werk? | □ nee  □ ja |
|  | Zwelling in mijn handen | □ nee  □ ja → | blijft dit gevoel aanwezig in de uren na het werk? | □ nee  □ ja |
|  | Zwelling en drukpijn in de bovenste extremiteit | □ nee  □ ja |  |  |
|  | Chronische pijn en/of een doof/tintelend gevoel in de bovenste extremiteit | □ nee  □ ja |  |  |
|  | Ik heb de indruk dat in de bovenste extremiteit waarin ik klachten heb, veranderingen zijn opgetreden wat betreft kleur van huid, temperatuur en zweterigheid | □ nee  □ ja |  |  |
|  | Tijdens mijn werk gebruik ik hulpmiddelen in de werkomgeving zoals een muismatje, een documenthouder of een voetensteun om mijn klachten te verminderen. | □ nee  □ ja |  |  |
|  | Tijdens mijn werk gebruik ik hulpmiddelen zoals 'braces' of een spalk om mijn klachten te verminderen | □ nee  □ ja |  |  |
